# Asbestos awareness among the residents of St. Kitts and Nevis: a cross-sectional study

**DOI:** 10.1186/s12992-022-00874-w

**Published:** 2022-09-24

**Authors:** Denrick Jeffers, Yu-Chi Liao, Ken Takahashi, Ro-Ting Lin

**Affiliations:** 1 Department of Occupational Safety and Health, College of Public Health, China Medical University, Taichung, 406040 Taiwan; 2 Environmental Health Department, Ministry of Health, Basseterre, St. Kitts and Nevis; 3 Department of Psychology, College of Medical and Health Science, Asia University, Taichung, 41354 Taiwan; 4 Center for Prevention and Treatment of Internet Addiction, Asia University, Taichung, 41354 Taiwan; 5 Clinical Psychology Center, Asia University Hospital, Taichung, 41354 Taiwan; 6 School of Population and Global Health, University of Western Australia, Perth, WA 6009 Australia; 7 University of Occupational and Environmental Health, Kitakyushu, Fukuoka, 807-8555 Japan; 8 Asbestos Diseases Research Institute, Concord, Sydney 2139 Australia

**Keywords:** Asbestos, Awareness, Health literacy, Asbestos ban, Asbestos-related diseases, St. Kitts and Nevis

## Abstract

**Background:**

High levels of public awareness regarding the hazards of asbestos, rights to health, and benefits of an asbestos-free country can increase advocacy and political commitment to a total ban on asbestos. We aimed to investigate asbestos awareness and associated sociodemographic characteristics among the adult population of St. Kitts and Nevis.

**Methods:**

In this cross-sectional study, 1009 participants completed an online questionnaire with questions about sociodemographic data and asbestos awareness. We applied multiple regression models to estimate associations between sociodemographic factors, levels of asbestos knowledge, and attitudes toward asbestos management.

**Results:**

We found that 70% of residents of St. Kitts and Nevis considered asbestos exposure to be a general public concern and believed the government should prevent it. Of all participants, 54% were in favor of completely banning the use and importation of all asbestos products and materials; those with higher levels of asbestos knowledge were more likely to favor a total ban. Higher proportions and odds of favoring a total asbestos ban were also observed in participants aged ≥ 30 years, women, those with higher education, and those living in St. Kitts (*vs.* Nevis).

**Conclusions:**

These findings support implementing policies to regulate and outright ban the use of asbestos products and materials in St. Kitts and Nevis. This data can be used to develop tailored campaigns to improve asbestos knowledge among sociodemographic groups with lower asbestos awareness, such as in the wider Caribbean and other under-resourced countries.

**Supplementary Information:**

The online version contains supplementary material available at 10.1186/s12992-022-00874-w.

## Background

Asbestos is a collective term for six types of fibrous minerals, which could be divided into serpentine (i.e., chrysotile [also known as white asbestos]) and amphibole (i.e., actinolite, amosite [also known as brown asbestos], anthophyllite, crocidolite [also known as blue asbestos], and tremolite) [1]. Asbestos fibers have many properties for industrial and commercial use, such as high tensile strength, flexibility to be woven, thermal stability, insulation, adsorption capacity, and resistance to chemical, thermal and biological degradation [1, 2]. However, the widespread use of asbestos and asbestos-containing products has exposed both workers and the general population to asbestos fibers in the workplace and the living environment [3].

Environmental and occupational exposure to asbestos may cause deaths from asbestos-related diseases [4]. Countries with high asbestos consumption deal with epidemics of asbestos-related diseases, typically 30 to 40 years after exposure [5, 6]. Many asbestos-related deaths could have been prevented if there were national bans on importing, exporting, and using asbestos [7]. Despite overwhelming evidence regarding the health implications of asbestos exposure and the best solutions to eliminate asbestos-related diseases, many countries still allow the use of asbestos and asbestos-containing products. More than two-thirds of countries still have not banned the use of asbestos [8].

The implementation of national policies for a total asbestos ban requires political commitments to obtain resources and support [9, 10]. High levels of public awareness of the hazards of asbestos, rights to health, and benefits of an asbestos-free country can influence advocacy and increase political commitments to a total ban on asbestos [11–14]. An enhanced level of awareness of the health implications of asbestos may be an impetus for people to start lobbying for policies geared at asbestos exposure prevention [15]. Hence, there should be campaigns and policies to increase public awareness about the risks of asbestos exposure and ways to manage asbestos-containing materials [16]. Radio talk shows, billboards, posters, fact sheets, television programs, and other social media platforms could be used for national asbestos awareness campaigns. These campaigns may inspire community leaders, health professionals, and other concerned persons to influence politicians and other legislative experts to initiate some well-needed asbestos exposure prevention policies [17]. However, the hurdles in translating scientific evidence into health prevention policy and multisectoral interventions to reduce the impact of asbestos-related diseases have been difficult to overcome, especially in low-income countries and countries in economic transition [18, 19].

St. Kitts and Nevis, located in the Caribbean, is the smallest country by land mass in the western hemisphere and has a population of approximately 54,500 [20]. Due to a lack of natural resources, its economy highly depends on tourism. St. Kitts and Nevis also generates substantial revenue from its Citizenship by Investment Program—a program that aggregates large budget inflows from the industry. As of 2014, budget inflows of the program represented 14% of the country’s gross domestic product (GDP); subsequently, the high income of the program helped clear the country’s debt burden, from 185% of GDP in 2009 to 60% of GDP in 2018 [21]. The St. Kitts and Nevis Citizenship by Investment Program attracts a high volume of clientele from foreign investors, such as China, home to the largest consumer market for second citizenship [22]. Foreign investors can obtain St Kitts and Nevis citizenship by making a certain amount of non-refundable charity donations or by investing in real estate. Foreign investors may also participate in large-scale construction projects on the islands. Many raw materials used in construction projects are likely to be sourced from foreign investors’ home countries, such as asbestos-containing cement, insulating boards, drywalls, and tiles. St. Kitts and Nevis allows trading of certain hazardous materials, such as asbestos-containing products; hence, residents have been exposed to asbestos products and materials for a long time. Approximately 82 metric tons of asbestos-containing products were exported to St. Kitts and Nevis between 2007 and 2020 [23]. These products were mainly fabricated asbestos fibers (55%) and asbestos cement (44%) [23]. The largest exporter was the United States (89%), followed by China (11%) [23]. The European Union and other countries, such as Australia, Canada, Denmark, and Jamaica, also exported small amounts of asbestos-containing products (< 1%) to St. Kitts and Nevis during this period [23].

There are two international conventions that are especially relevant to asbestos management: (1) the International Labour Organization (ILO) Convention No. 162 concerning Safety in the Use of Asbestos and (2) the Basel Convention on the Control of Transboundary Movements of Hazardous Wastes and their Disposal [10]. Unfortunately, St. Kitts and Nevis has yet to ratify any of these two conventions. St. Kitts and Nevis has yet to develop guidelines and regulations for asbestos and asbestos-related diseases related to occupational exposure limits. Currently, there are no specific guidelines or regulations for the use, removal, or disposal of asbestos materials. In addition, the country has no locally available mechanism to diagnose mesothelioma or other asbestos-related diseases, and there are no available government data or statistics, from online databases or official documents, on the incidence, prevalence, or mortality of asbestos-related diseases. Given that St. Kitts and Nevis has not banned asbestos, asbestos and asbestos-containing materials can be continually imported and used without concern for asbestos exposure prevention. There are no exposure limits for asbestos to help workers and residents become more aware of asbestos and asbestos-related diseases.

This lack of awareness and data on asbestos hazards is a potential reason for the continued use of asbestos in vulnerable countries such as St. Kitts and Nevis. Awareness campaigns with strategically designed messages with a clear aim and intent have been shown to influence attitudes and behaviors in public health and medicine [24]. Assessing public awareness is important in identifying gaps and strengthening prevention efforts. Therefore, this study aimed to investigate the awareness of asbestos among the residents of St. Kitts and Nevis. We used an online questionnaire to collect participants’ awareness of asbestos exposure, health risks, and preventive measures against asbestos exposure.

Few studies have been published on the non-occupational awareness level of asbestos among the residents of many countries, especially developing countries. Studies of factors that may affect the asbestos awareness level of participating workers have been published. A recently conducted survey of asbestos containing materials in the brewing industry in Ghana revealed that the level of education was significantly associated with the level of awareness on asbestos containing materials in the workplace; the level of awareness of asbestos containing materials presence in the brewery facilities among workers was very limited [25]. A study on asbestos awareness amongst licensed electricians in Malta revealed that 74% of the respondents in that survey admitted lacking knowledge of the health risks associated with exposure to asbestos dust, and 90% of the respondents had never heard about mesothelioma [26]. In a survey of construction workers in Turkey, 70.4% of the workers had no information about asbestos [27]. In another study conducted in a region of Turkey, the knowledge and awareness level of asbestos was low in 70% of participants [28]. Most studies have not focused on levels of asbestos awareness of the general public. In this study we aimed to investigate asbestos awareness and associated sociodemographic characteristics in St. Kitts and Nevis.

## Methods

### Participants

The study participants were citizens of St. Kitts and Nevis. For this population-based cross-sectional study, adult resident citizens were recruited randomly via the internet. As of November 27, 2020, approximately 81% of the population in St. Kitts and Nevis could access the internet at home via a computer or mobile device and connection [29]. Thus, we had the potential to reach four out of every five persons within the federation of St. Kitts and Nevis. Assuming an expected proportion of 30% of the population having sufficient knowledge and awareness about asbestos, based on a previously published study and a 5% level of significance [28, 30], the required sample size was calculated to be 322. Respondents who answered that they were citizens, aged ≥ 18 years, and currently residing in the country could continue the questionnaire. Anyone who answered that they were < 18 years, declined to participate or withdrew from completing the questionnaire, or who stated that they were non-resident citizens, non-citizen residents, or visitors in St. Kitts and Nevis were prevented from completing the questionnaire.

### Ethics review

This study was approved by St. Kitts & Nevis Interim Ethics Review Committee (IERC-2021–03-045) and the Central Regional Ethics Committee of China Medical University, Taiwan (CRREC-110–038).

### Setting and duration

Due to the COVID-19 pandemic and social distancing protocols, the questionnaire was administered online using Google Forms. All potential participants were recruited by a network of persons sharing the survey URL on WhatsApp, Messenger, and Facebook. The questionnaire link provided an informed consent statement covering the rights of each potential participant and a summary of the research study on the first page. Contact information was provided in the event that the participant wanted to ask questions and clear up any misunderstandings or doubts that they might have concerning the research study. Thus, before anyone could agree to participate in the research study, they would have read the “Research Study Information Letter/Informed Consent.”

After agreeing to participate and providing informed consent, participants were asked to complete a two-part questionnaire (Additional file 1). The first part of the questionnaire included sociodemographic questions, including age, gender (male or female), education level, residential area (St. Kitts or Nevis), and occupation. The second part of the questionnaire comprised ten questions about asbestos knowledge and attitudes. The survey was launched on April 26, 2021, and lasted 80 days, closing on July 15, 2021. A total of 1089 responses were received. We used email addresses to identify each participant, and for multiple responses with the same email address, only the first response was included in the analysis. After excluding duplicates and incomplete responses, the total sample size was 1009.

### Asbestos questionnaire

We used ten questions to assess asbestos awareness (see Additional file 1): seven questions measuring the level of asbestos knowledge and three questions assessing attitudes toward asbestos management. The total asbestos knowledge score ranged from 0 to 7, based on the number of correct answers to the seven questions (see Additional file 2). A higher score represented a higher level of asbestos knowledge. The questionnaire content was assessed through critique by expert specialists and a pilot questionnaire was conducted with a sample of 13 participants. The Cronbach’s alpha of the pilot questionnaire was 0.795. In the final sample of 1009 participants, the Cronbach’s alpha of the questionnaire was 0.815. The intra-class correlation coefficient was 0.815 (95% confidence interval [CI]: 0.797–0.831).

### Analysis

Means and standard deviations were calculated to describe the average and variability of the levels of asbestos knowledge in each sociodemographic group. Proportions were used to describe the distribution of attitudes toward asbestos management in each sociodemographic group. A multiple linear regression model was applied to determine associations between the levels of asbestos knowledge and the demographic, educational, occupational, and residential variables. Multiple logistic regression models were used to estimate odds ratios (ORs) and their 95% CIs to describe the probability of attitudes toward asbestos management in participants with specific characteristics compared to those without.

## Results

The mean level of asbestos knowledge among all 1009 participants was 3.65. Table 1 shows the number of participants and mean level of asbestos knowledge in each sociodemographic group. Multiple linear regression models were adjusted for age group, gender, education level, residential area, and occupation. All variables except gender showed significant associations with the level of asbestos knowledge. By sociodemographic group, the level of asbestos knowledge was higher in people aged ≥ 30 years than those aged < 30 years (means 3.92–3.96 vs. 2.82; *p* < 0.0001), in Kittitians than in Nevisians (mean 3.76 vs. 3.30; *p* = 0.0139), and in people working in the government than those working in the private sector (mean 4.02 vs. 3.40; *p* = 0.0043). In addition, the level of asbestos knowledge increased with education level: from some secondary (mean 2.31), to secondary (mean 3.40), some tertiary (mean 3.82), and university (mean 4.37) (*p* < 0.0001). There was no significant difference in the level of asbestos knowledge by gender.

**Table 1:** Participants’ sociodemographic characteristics and level of asbestos knowledge

All participants	***n* (%)**		**Mean level of asbestos knowledge (SD)**		Estimation of level of asbestos knowledge in regression model^*^		
					***β***	**95% CI**	***p* value**
	1009	(100%)	3.65	(2.01)	-	-	-
Age group							
< 30 years	261	(25%)	2.82	(2.13)	0	(reference)	
30–39 years	350	(35%)	3.92	(1.78)	0.217	(0.066, 0.367)	< 0.0001
40–49 years	241	(24%)	3.96	(1.88)	0.210	(0.045, 0.374)	< 0.0001
≥ 50 years	157	(16%)	3.92	(2.13)	0.219	(0.033, 0.405)	< 0.0001
Gender							
Women	634	(63%)	3.63	(1.98)	0	(reference)	
Men	375	(37%)	3.67	(2.07)	0.015	(-0.105, 0.135)	0.6137
Education level							
Some secondary	98	(10%)	2.31	(2.20)	0	(reference)	
Completed secondary	362	(36%)	3.40	(1.98)	0.252	(0.039, 0.465)	< 0.0001
Some tertiary	321	(32%)	3.82	(1.98)	0.329	(0.114, 0.545)	< 0.0001
Completed university	228	(22%)	4.37	(1.63)	0.391	(0.163, 0.619)	< 0.0001
Residential area							
St. Kitts	757	(75%)	3.76	(1.96)	0	(reference)	
Nevis	252	(25%)	3.30	(2.12)	-0.073	(-0.207, 0.061)	0.0139
Occupation							
Government	408	(40%)	4.02	(1.85)	0	(reference)	
Private	601	(60%)	3.40	(2.08)	-0.086	(-0.206, 0.034)	0.0043

Abbreviation: *β* parameter estimate, *CI* confidence interval, *n* number of participants, *SD* standard deviation

^*^ Estimated using multiple linear regression model, adjusted for all listed independent variables (age group, gender, education level, residential area, and occupation)

Over 70% of participants considered that the general public should be concerned about asbestos and that it was the government’s duty to prevent asbestos exposure (71%; Table 2). After adjusting for all variables in the multiple logistic regression models, the odds of agreeing that the general public should be concerned about asbestos were 1.54–1.59-fold higher among people aged ≥ 40 years than those aged < 30 years. The odds of agreeing that it was the government’s duty to prevent asbestos exposure were 1.82-fold higher among people aged ≥ 50 years than those aged < 30 years (OR 1.82; 95% CI: 1.15–2.89) and 2.05-fold higher among people with a university education than among those with some secondary education (OR 2.05; 95% CI: 1.16–3.62).

**Table 2:** Associations between sociodemographic variables (independent variables) and asbestos attitudes (dependent variable)

	***n***	**The general public should be concerned about asbestos exposure**					**It is the government’s duty to prevent asbestos exposure**					**Asbestos ban is the best option to prevent asbestos exposure**				
		Agree (%)	OR^*^	95% CI^*^	OR†	95% CI^†^	Agree (%)	OR^*^	95% CI^*^	OR^†^	95% CI^†^	Agree (%)	OR^*^	95% CI^*^	OR†	95% CI^†^
All participants	1009	711 (70%)	-	-	-	-	717 (71%)	-	-	-	-	549 (54%)	-	-	-	-
Age group																
< 30 years	261	166 (64%)	1.00	(reference)	1.00	(reference)	168 (64%)	1.00	(reference)	1.00	(reference)	108 (41%)	1.00	(reference)	1.00	(reference)
30–39 years	350	253 (72%)	1.42	(0.99–2.01)	0.89	(0.60–1.32)	259 (74%)	1.44	(1.01–2.06)	1.09	(0.75–1.59)	196 (56%)	1.64	(1.17–2.30)	1.15	(0.79–1.66)
40–49 years	241	177 (73%)	1.54	(1.04–2.26)	0.95	(0.61–1.46)	169 (70%)	1.26	(0.86–1.86)	0.94	(0.63–1.41)	147 (61%)	2.23	(1.53–3.25)	1.60	(1.06–2.41)
≥ 50 years	157	115 (73%)	1.59	(1.02–2.47)	0.88	(0.54–1.45)	121 (77%)	1.82	(1.15–2.89)	1.29	(0.79–2.09)	98 (62%)	2.49	(1.62–3.81)	1.62	(1.01–2.59)
Gender																
Women	634	462 (73%)	1.00	(reference)	1.00	(reference)	462 (73%)	1.00	(reference)	1.00	(reference)	360 (57%)	1.00	(reference)	1.00	(reference)
Men	375	255 (68%)	0.90	(0.68–1.19)	0.85	(0.62–1.17)	255 (68%)	0.84	(0.63–1.12)	0.82	(0.61–1.11)	189 (50%)	0.75	(0.57–0.99)	0.70	(0.52–0.94)
Education level																
Some secondary	98	66 (67%)	1.00	(reference)	1.00	(reference)	68 (69%)	1.00	(reference)	1.00	(reference)	44 (45%)	1.00	(reference)	1.00	(reference)
Completed secondary	362	254 (70%)	1.09	(0.66–1.78)	0.60	(0.34–1.05)	226 (62%)	0.80	(0.49–1.31)	0.56	(0.33–0.96)	148 (41%)	0.83	(0.52–1.33)	0.47	(0.28–0.80)
Some tertiary	321	219 (68%)	0.96	(0.58–1.59)	0.43	(0.24–0.76)	236 (74%)	1.30	(0.78–2.18)	0.85	(0.49–1.47)	188 (59%)	1.71	(1.06–2.76)	0.92	(0.54–1.57)
Completed university	228	172 (75%)	1.30	(0.76–2.23)	0.45	(0.24–0.85)	187 (82%)	2.05	(1.16–3.62)	1.16	(0.63–2.13)	169 (74%)	3.25	(1.92–5.49)	1.51	(0.84–2.69)
Residential area																
St. Kitts	757	543 (72%)	1.00	(reference)	1.00	(reference)	528 (70%)	1.00	(reference)	1.00	(reference)	430 (57%)	1.00	(reference)	1.00	(reference)
Nevis	252	168 (67%)	0.79	(0.58–1.08)	0.93	(0.66–1.31)	189 (75%)	1.31	(0.94–1.83)	1.49	(1.05–2.11)	119 (47%)	0.67	(0.49–0.91)	0.74	(0.53–1.03)
Occupation																
Government	408	303 (74%)	1.00	(reference)	1.00	(reference)	305 (75%)	1.00	(reference)	1.00	(reference)	244 (60%)	1.00	(reference)	1.00	(reference)
Private	601	408 (68%)	0.82	(0.62–1.08)	1.01	(0.73–1.40)	412 (69%)	0.84	(0.63–1.13)	0.95	(0.70–1.28)	305 (51%)	0.87	(0.66–1.15)	1.02	(0.76–1.37)
Level of asbestos knowledge, per 1-level increment	1009	-	-	-	1.66	(1.53–1.81)		-	-	1.35	(1.25–1.46)		-	-	1.58	(1.45–1.71)

Abbreviations: *β* parameter estimate, *CI* confidence interval, *n* number of participants, *OR* odds ratio

^*^Adjusted for age group, gender, education level, residential area, and occupation

^†^Adjusted for age group, gender, education level, residential area, occupation, and level of asbestos knowledge

As for the best option to prevent asbestos exposure, 54% of participants chose a total asbestos ban (Table 2). However, there were significant differences among sociodemographic groups in attitudes toward an asbestos ban. Higher proportions and odds of selecting a total asbestos ban were observed among those who were ≥ 30 years, women, those with higher education, and those who lived in St. Kitts.

People with higher levels of asbestos knowledge had higher odds of selecting the following answers to the questionnaire: (a) the general public should be concerned about asbestos (OR 1.66; 95% CI: 1.53–1.81), (b) the government is responsible for preventing asbestos exposure (OR 1.35; 95% CI: 1.25–1.46), and (c) a total asbestos ban is the best option for preventing exposure (OR 1.58; 95% CI: 1.45–1.71).

## Discussion

To the best of our knowledge, this is the first study to investigate asbestos awareness and associated sociodemographic characteristics in St. Kitts and Nevis. The findings of this study may provide useful information to policymakers about the level of asbestos awareness in St. Kitts and Nevis. The findings may also be used to inform government, public health officials, and other stakeholders on public health interventions, awareness, and policy initiatives on asbestos.

In this cross-sectional national online survey, we investigated the level of asbestos knowledge and attitudes of 1009 adults. We found that > 70% of participants considered asbestos exposure to be a general public concern and that it was the duty of the government to prevent it. However, only 54% of the respondents considered a total asbestos ban to be the best option to prevent asbestos exposure. Supporters of a total asbestos ban were those with a higher level of asbestos knowledge. As reflected in the average score of asbestos knowledge of 3.65, the respondents answered an average of 52.1% of questions correctly regarding asbestos hazards, exposure scenarios, and adverse health outcomes. The lack of sufficient asbestos knowledge may impede campaigns for an asbestos ban even where asbestos exposure is already a public concern.

However, a high proportion (70%) of respondents agreed that asbestos exposure is a general public concern in St. Kitts and Nevis. A similarly high proportion (71%) of respondents agreed that it was the government’s duty to prevent asbestos exposure. In most countries, the health sector represents a critical area of government responsibility [31]. Government health departments and related agencies are responsible for monitoring, protecting, and improving the health of residents and citizens [31]. Many residents and citizens of the country of St. Kitts and Nevis also regard the government as the responsible party for protecting people’s health and welfare [32]. Residents in St. Kitts and Nevis may also feel that the government has the resources and expertise to identify, remove, and prevent asbestos exposure, whereas the general public does not have that expertise.

In our question regarding the best option to avoid asbestos exposure (question 10), the answer choices were as follows: (a) to ban the use and import of all asbestos products and materials from entering St. Kitts and Nevis, (b) to wear personal protective equipment, and (c) I do not know. Whereas the majority of respondents agreed that asbestos exposure was a general public concern and that its prevention was the duty of the government, only 54% of participants selected a total asbestos ban as the best option to prevent asbestos exposure. Of the remaining participants, 13% selected wearing personal protective equipment as the best option, and 33% selected “I do not know.” This finding suggests that people may lack awareness of the pros and cons of potential solutions. They may not know anyone who has been diagnosed with an asbestos-related disease. Overlooking solutions to prevent asbestos exposure could also be due to scarcity or lack of epidemiological data and diagnosis of asbestos-related diseases [12]. Even among people who are aware of asbestos hazards, the cost, endurance, and performance of alternatives to asbestos may guide people away from selecting an asbestos ban as the best option [33]. However, our findings showed that a higher level of asbestos knowledge was associated with attitudes toward an asbestos ban: people with higher levels of asbestos knowledge were more likely to select a total ban as the best option to eliminate exposure risks. More asbestos knowledge could help people understand the difference between banning asbestos and wearing personal protective equipment as solutions to preventing asbestos-related diseases.

We found that the level of asbestos knowledge was significantly related to age, education, residential area, and occupation. Specifically, higher levels of asbestos knowledge were found in people aged ≥ 30 years, with higher education, living in St. Kitts, and working in the government. While age was a significant predictor in our study, it was not found significant in a previous study in Turkey [28]. However, age is related to the accessibility of higher education in St. Kitts and Nevis. University enrollment is expensive for most Kittitians and Nevisians, and it is difficult to attain higher levels of education until later years in life, when one can have saved up enough money to secure a loan or to pay for university-level education overseas or through distance learning via the internet. Meanwhile, our findings that education level and residential area are significant factors are consistent with the Turkish study, which recruited 760 hospital patients and their relatives and reported a higher level of asbestos knowledge and awareness among those who lived in an urban area, had graduated from a university, and were employed or retired [28].

Education level is among the most important predictors of health literacy [34, 35]. Higher educational attainment represents greater ability to understand, search for, and acquire scientific information and skills. Health literacy is also higher among people living in urban areas than those in rural areas [36]. Given that St. Kitts is the legislative and commercial capital, it is considered more urban than Nevis. Another potential factor in our finding that Kittitians were more knowledgeable and aware of asbestos was a number of news reports related to suspected asbestos exposure in government institutions (e.g., National Information and Communications Technology Centre) [37], schools (e.g., Irishtown Primary School and Cayon High School) [38], and public facilities (e.g., water pipes). For example, on Saturday, April 26, 2014, the Ministry of Education released a statement to inform parents and guardians of students attending the Basseterre High School in St. Kitts that there would be no school on Monday, April 28 [39]. The temporary closure of the secondary school was speculated to be due to asbestos contamination, which became a well-publicized issue in this region [40]. In another incident, the government announced and implemented an island-wide project on St. Kitts in 2019 to replace the asbestos pipes in the existing water transportation system with polyvinyl chloride pipes [41]. It is likely due to these occasional events of suspected asbestos exposure in government buildings, institutions, and facilities that the government workers in our sample were generally more aware of asbestos compared with people working in the private sector.

We did not find a significant gender difference in the participants’ responses to the questions about whether the general public should be concerned about asbestos in their daily lives or whether it is the government’s duty to prevent asbestos exposure. However, we did find a significant gender difference in answers to the question concerning the best option to prevent asbestos exposure. A higher proportion of women than men agreed that the best option for asbestos exposure was a total asbestos ban. Women are more experienced with changes in the living environment (e.g., needing to manage resources), which leads them to be more concerned about environmental hazards and more willing to adopt environmental behaviors (e.g., being plastic-free) and actively engage in environmental health issues [42]. St. Kitts and Nevis is a matriarchal society, and nearly half of all households (47%) are headed by single women [43]. The eventual closure of the asbestos-contaminated school in St. Kitts may have heightened parents’, and particularly mothers’, concerns about potential asbestos exposure in schools.

Now is the time to raise awareness regarding asbestos hazards and ban asbestos in St. Kitts and Nevis. The first policy recommended by the World Health Organization for the elimination of asbestos-related diseases is to stop the use of all types of asbestos [44]. Banning asbestos production, export, and import would significantly reduce asbestos-related mortality globally, not only in occupational settings but also in households and other non-occupational settings [45]. St. Kitts and Nevis does not produce asbestos. Therefore, the government of St. Kitts and Nevis should proactively fight against the incidence and mortality of asbestos-related diseases, including adopting and ratifying the ILO Convention No. 162 and the Basel Convention as well as conducting extensive asbestos exposure prevention awareness campaigns.

There were a number of limitations in our study. First, the study design was cross-sectional, and causal conclusions should not be inferred. Second, participants completed questionnaires anonymously via the internet, and we had difficulty reaching certain types of participants, such as adults without internet access, who are illiterate, and who are older and have low technological literacy. This may have led to selection bias, with more health-oriented and more conscientious respondents opting to participate in the study. If so, this limitation could mean reduced generalizability of the results to the general population. In the initial stages of study development, we had planned to conduct formal in-person questionnaire interviews, but when the COVID-19 pandemic occurred, many governments, including St. Kitts and Nevis, imposed movement restrictions. These were not limited to international air travel but also included “stay-at-home” and social distancing policies. We had no choice but to implement and conduct the survey online. Third, due to the questionnaire structure, participants were not required to explain their choice of not wanting to ban asbestos imports. However, we could recognize that residents with higher levels of asbestos knowledge had higher odds of selecting a total asbestos ban and vice versa. This finding indicates that participants lacking knowledge of asbestos would not select banning asbestos as the best option for preventing exposure.

Currently, the political will and policies of the country have not been geared toward an asbestos ban. Many people are unaware of the intangible costs of asbestos, especially asbestos-related diseases and death. The lack of appropriate tools and equipment to identify asbestos and asbestos-related diseases hinders promotion of the asbestos hazard. Small and under-resourced countries engaging in international trade are especially vulnerable to economic pressure from powerful asbestos-exporting countries. The failure of countries to ban asbestos reflects the enormous power of the asbestos industry and its political allies who act against the best interests of public health and place short-term profits ahead of human well-being [46, 47].

## Conclusions

Residents of St. Kitts and Nevis consider asbestos exposure to be a general public concern and to be the government’s duty to prevent. Over half of the population favor a complete ban on the use and importation of all asbestos products and materials, and this is the common view of those with a high level of asbestos knowledge. These findings support implementing policies that will regulate and outright ban the use of asbestos products and materials in St. Kitts and Nevis, and can also be used to develop campaigns to improve asbestos knowledge that are tailored for sociodemographic groups with lower levels of awareness in St. Kitts and Nevis, the wider Caribbean, and other under-resourced countries.

## Supplementary Information

**Additional file 1.** Asbestos awareness questionnaire

**Additional file 2.** Scoring for asbestos knowledge

## Data Availability

The datasets used and/or analyzed during the current study are available from the corresponding author on reasonable request.

## Abbreviations

CI: Confidence interval
OR: Odds ratio

## References

[1] Virta RL. Asbestos: geology, mineralogy, mining, and uses. U.S. Geological Survey 2002.

[2] IARC Working Group on the Evaluation of Carcinogenic Risks to Humans. A review of human carcinogens. Part C: Arsenic, metals, fibres and dusts. Lyon, France: International Agency for Research on Cancer; 2012. PMC4781271 23189751

[3] Zeber-Dzikowska I, Wojciechowska M, Dziechciaz M, Gietka M, Czarny-Dzialak M, Grudzien R, et al. Environmental and occupational exposure to asbestos as an ongoing challenge for environmental and health education. J Elem. 2022;27(1).

[4] Stayner L, Welch LS, Lemen R (2013). The worldwide pandemic of asbestos-related diseases. Annu Rev Public Health.

[5] Lin R-T, Takahashi K, Karjalainen A, Hoshuyama T, Wilson D, Kameda T (2007). Ecological association between asbestos-related diseases and historical asbestos consumption: an international analysis. Lancet.

[6] Rath EM, Yuen ML, Odgerel C-O, Lin R-T, Soeberg M, Nowak AK (2022). The ecological association between asbestos consumption and asbestos-related diseases 15 years later. Environ Health Perspect.

[7] Arachi D, Soeberg M, Chimed-Ochir O, Lin R-T, Takahashi K. Trend in the global incidence of mesothelioma: is there any changing trend after asbestos regulation and ban? In: Nakano T, Kijima T, editors. Malignant pleural mesothelioma. 1 ed. Singapore: Springer; 2021.

[8] Kazan-Allen L. Current asbestos bans. July 15, 2019 [cited February 10, 2022]. Available from: http://www.ibasecretariat.org/alpha_ban_list.php.

[9] Wu  HY-J, Lin  R-T, Wang J-D , Cheng  Y (2017). Transnational dynamics amid poor regulations: Taiwan’s asbestos ban actions and experiences. Int J Environ Res Public Health.

[10] Lin R-T, Chien L-C, Jimba M, Furuya S, Takahashi K (2019). Implementation of national policies for a total asbestos ban: a global comparison. Lancet Planet Health.

[11] Frieden TR (2010). A framework for public health action: the health impact pyramid. Am J Public Health.

[12] Bahk J, Choi Y, Lim S, Paek D (2013). Why some, but not all, countries have banned asbestos. Int J Occup Environ Health.

[13] Glass WI, Armstrong R, Chen G (2017). Banning asbestos in New Zealand, 1936–2016, an 80-year long saga. Int J Environ Res Public Health.

[14] Yoon YR, Kwak KM, Choi Y, Youn K, Bahk J, Kang DM (2018). The asbestos ban in Korea from a grassroots perspective: why did it occur?. Int J Environ Res Public Health.

[15] Phillips GA, Lindgren MKM (2010). The Australian Asbestos Network–how journalism can address a public health disaster. Observatorio (OBS*).

[16] Soeberg M, Vallance DA, Keena V, Takahashi K, Leigh J (2018). Australia's ongoing legacy of asbestos: significant challenges remain even after the complete banning of asbestos almost fifteen years ago. Int J Environ Res Public Health.

[17] Ruff K (2017). How Canada changed from exporting asbestos to banning asbestos: the challenges that had to be overcome. Int J Environ Res Public Health.

[18] LaDou J, Castleman B, Frank A, Gochfeld M, Greenberg M, Huff J (2010). The case for a global ban on asbestos. Environ Health Perspect.

[19] Algranti E, Ramos-Bonilla JP, Terracini B, Santana VS, Comba P, Pasetto R (2019). Prevention of asbestos exposure in Latin America within a global public health perspective. Ann Glob Health.

[20] Central Intelligence Agency. The World Factbook. Saint Kitts and Nevis 2022 [cited June 11, 2022]. Available from: https://www.cia.gov/the-world-factbook/countries/saint-kitts-and-nevis/.

[21] Cover-Kus H. Weighing up second passport power in small states: a SWOT analysis of the citizenship-by-investment industry: Commonwealth Secretaria; 2019 [cited August 1, 2022]. Available from: https://www.thecommonwealth-ilibrary.org/index.php/comsec/catalog/view/423/423/3771.

[22] Surak K. Global citizenship 2.0: the growth of citizenship by investment programs. 2016.

[23] World Bank. World integrated trade solutions Washington, D.C., United States [cited July 29, 2022]. Available from: https://wits.worldbank.org/.

[24] Kidd LR, Garrard GE, Bekessy SA, Mills M, Camilleri AR, Fidler F (2019). Messaging matters: a systematic review of the conservation messaging literature. Biol Conserv.

[25] Nunoo E, Panin A, Essien B (2018). Environmental health risk assessment of asbestos-containing materials in the brewing industry in Ghana. J Environ Res.

[26] Salomone S. Asbestos awareness amongst licensed electricians in Malta. 2010.

[27] Konak Ö, Tatlıpınar ME, Omurtag GZ. Evaluation of knowledge level about asbestos exposure in urban transformation construction areas. Acta Pharm Sci. 2017.

[28] Metintas S, Ak G, Bogar F, Yilmaz S, Metintas M (2017). Asbestos knowledge and awareness level in central part of Anatolia. Int J Occup Environ Health.

[29] Bleeker A. Strengthening ICT and knowledge management capacity in support of the sustainable development of multi-island Caribbean SIDS. Santiago, Chile: Economic Commission for Latin America and the Caribbean (ECLAC); 2019.

[30] Charan JBT (2013). How to calculate sample size for different study designs in medical research?. Indian J Psychol Med.

[31] Al-Dmour H, Masa'deh R, Salman A, Abuhashesh M, Al-Dmour R (2020). Influence of social media platforms on public health protection against the COVID-19 pandemic via the mediating effects of public health awareness and behavioral changes: integrated model. J Med Internet Res.

[32] Snyder A, Matthew S, Leahy N, Gaul R, Hood TL, Hijmans K (2022). Island communities and disaster resilience: applying the EnRiCH community resilience framework. Public Health Nurs.

[33] Li JH, Dong QY, Yu KL, Liu LL (2014). Asbestos and asbestos waste management in the Asian-Pacific region: trends, challenges and solutions. J Clean Prod.

[34] van der Heide I, Wang J, Droomers M, Spreeuwenberg P, Rademakers J, Uiters E (2013). The relationship between health, education, and health literacy: results from the Dutch Adult Literacy and Life Skills Survey. J Health Commun.

[35] Stormacq C, Van den Broucke S, Wosinski J (2019). Does health literacy mediate the relationship between socioeconomic status and health disparities?. Integrative review Health Promot Int.

[36] Aljassim N, Ostini R (2020). Health literacy in rural and urban populations: a systematic review. Patient Educ Couns.

[37] Ferlance J. Officials dispel rumours of asbestos threat at government institutions: www.SKNvibes.com; April 25, 2012 [cited May 6, 2022]. Available from: https://www.sknvibes.com/news/newsdetails.cfm/56989.

[38] Ferlance J. Asbestos find leaves Cayon High building to be demolished: www.SKNvibes.com; December 6, 2012 [cited May 6, 2022]. Available from: https://www.sknvibes.com/news/newsdetails.cfm/66835.

[39] St Kitts Nevis Observer. BHS closed relocation options being examined by R. Wilson. April 25, 2014 [cited June 9, 2022]. Available from: https://www.thestkittsnevisobserver.com/bhs-closed-relocation-options-being-examined-by-r-wilson/.

[40] Ferlance J. Infestation at Basseterre High School: www.SKNVibes.com; November 19, 2012 [cited May 3, 2022]. Available from: https://www.sknvibes.com/news/newsdetails.cfm/66078.

[41] St. Kitts Nevis Information Service. Government intends to relocate water-lines from beneath island main road. July 31, 2019 [cited January 25, 2021]. Available from: https://www.sknis.gov.kn/2019/07/31/government-intends-to-relocate-water-lines-from-beneath-island-main-road/.

[42] Shepherd A, Jepson R, Watterson A, Evans JMM (2012). Risk perceptions of environmental hazards and human reproduction: a community-based survey. ISRN Public Health.

[43] Drzewiecki DM, Wavering HM, Milbrath GR, Freeman VL, Lin JY. The association between educational attainment and resilience to natural hazard-induced disasters in the West Indies: St. Kitts & Nevis. Int J Disaster Risk Reduct. 2020;47:101637.

[44] World Health Organization. Asbestos: elimination of asbestos-related diseases. February 15, 2018 [cited August 5, 2022]. Available from: https://www.who.int/news-room/fact-sheets/detail/asbestos-elimination-of-asbestos-related-diseases.

[45] World Health Organization. Elimination of asbestosrelated diseases 2014 [cited August 5, 2022]. Available from: https://www.who.int/publications/i/item/WHO-FWC-PHE-EPE-14.01.

[46] Lemen RA, Landrigan PJ (2017). Toward an asbestos ban in the United States. Int J Environ Res Public Health.

[47] Terracini B (2019). Contextualising the policy decision to ban asbestos. Lancet Planet Health.

