# Supplementary material for: Asbestos awareness among the residents of St. Kitts and Nevis: a cross-sectional study
Source: Global Health. 2022 Sep 24;18:83. doi: 10.1186/s12992-022-00874-w (PMC9509556; doi:10.1186/s12992-022-00874-w)
Supplement: Supplementary file 1 — Additional file 1. Asbestos awareness questionnaire [file 12992_2022_874_MOESM1_ESM.docx]

# Additional file 1

# Asbestos awareness questionnaire

**Part 1. Demographics**

**Age group**

🞎 29 years or younger

🞎 30–39 years

🞎 40–49 years

🞎 50–59 years

🞎 60–69 years

🞎 70 years and older

**Gender**

🞎 Male

🞎 Female

**Education level**

🞎 Some secondary

🞎 Completed secondary

🞎 Some tertiary

🞎 Completed university

**Residential area**

🞎 St. Kitts

🞎 Nevis

**Occupation**

🞎 Trades (including mechanic, carpenter, welder, HVAC)

🞎 Leisure and Hospitality

🞎 Construction

🞎 Agriculture & Fishing

🞎 Manufacturing

🞎 Other private

🞎 Government

**Part 2. Asbestos Knowledge and Awareness Questionnaire**

**1. Have you ever heard of the term “asbestos” before today?**

🞎 Yes

🞎 No

🞎 I am not sure

**2. Which of the following statements best describe the term “asbestos”?**

🞎 Asbestos is a group of six naturally occurring minerals composed of soft and flexible fibers

🞎 Asbestos is a chemical that was created in the laboratory

🞎 I do not know

**3. Which of the following items may contain asbestos?**

🞎 Duct tape

🞎 Corrugated roof

🞎 Sprayed on form of insulation such as in ships

🞎 Vehicle brake pads

🞎 Pipe insulation

🞎 Ceiling tiles and/or floor tiles

🞎 Sheetrock

🞎 All of the above

🞎 None of the above

🞎 I do not know

**4. What is the primary and most common exposure route for asbestos to enter the human body?**

🞎 Ingestion

🞎 Dermal contact

🞎 Inhalation

🞎 Injection

**5. Which of the following diseases are related to asbestos exposure?**

🞎 Pleural plaque

🞎 Mesothelioma

🞎 Asbestosis

🞎 Lung cancer

🞎 All of the above

🞎 None of the above

🞎 I do not know

**6. Most symptoms of asbestos-related diseases start appearing within a certain time period after initial exposure. Which option best describes the latency period for asbestos related diseases?**

🞎 Less than 1 year

🞎 1–10 years

🞎 More than 10 years

🞎 I do not know

**7. Do you think that the general public in St. Kitts & Nevis should be concerned about asbestos exposure in their daily life?**

🞎 Yes

🞎 No

🞎 I do not know

**8. Which type of below exposure can cause disease after many years of asbestos?**

🞎 Occupational exposure

🞎 Environmental exposure, for example, neighborhood exposure

🞎 Household exposure, for example, washing clothes contaminated with asbestos

🞎 All of the above

🞎 None of the above

**9. Do you think that it is the government’s duty to prevent asbestos exposure in the daily life of the general population in St. Kitts & Nevis?**

🞎 Yes

🞎 No

🞎 I do not know

**10. Which of the following is the best option to prevent asbestos exposure in St. Kitts & Nevis?**

🞎 To ban the use and import of all asbestos products and materials from entering St. Kitts & Nevis

🞎 To wear personal protective equipment

🞎 I do not know
