# Supplementary material for: Asbestos awareness among the residents of St. Kitts and Nevis: a cross-sectional study
Source: Global Health. 2022 Sep 24;18:83. doi: 10.1186/s12992-022-00874-w (PMC9509556; doi:10.1186/s12992-022-00874-w)
Supplement: Supplementary file 2 — Additional file 2. Scoring for asbestos knowledge [file 12992_2022_874_MOESM2_ESM.docx]

# Additional file 2

# Scoring for asbestos knowledge

1. **Have you ever heard of the term “asbestos” before today?**

Yes = 1;

No = 0;

I am not sure = 0.

**2. Which of the following statements best describe the term “asbestos”?**

Asbestos is a group of six naturally occurring minerals composed of soft and flexible fibers = 1;

Asbestos is a chemical that was created in the laboratory = 0;

I do not know = 0.

**3. Which of the following items may contain asbestos?**

Duct tape = 0.5;

Corrugated roof = 0.5;

Sprayed on form of insulation such as in ships = 0.5;

Vehicle brake pads = 0.5;

Pipe insulation = 0.5;

Ceiling tiles and/or floor tiles = 0.5;

Sheetrock = 0.5;

All of the above = 1;

None of the above = 0;

I do not know = 0.

**4. What is the primary and most common exposure route for asbestos to enter the human body?**

Ingestion = 0;

Dermal contact = 0;

Inhalation = 1;

Injection = 0.

**5. Which of the following diseases are related to asbestos exposure?**

Pleural plaque = 0.5;

Mesothelioma = 0.5;

Asbestosis = 0.5;

Lung cancer = 0.5;

All of the above = 1;

None of the above = 0;

I do not know = 0.

**6. Most symptoms of asbestos-related diseases start appearing within a certain time period after initial exposure. Which option best describes the latency period for asbestos related diseases?**

Less than 1 year = 0;

1–10 years = 0;

More than 10 years = 1;

I do not know = 0.

**8. Which type of below exposure can cause disease after many years of asbestos?**

Occupational exposure = 0.5;

Environmental exposure, for example, neighborhood exposure = 0.5;

Household exposure, for example, washing clothes contaminated with asbestos = 0.5;

All of the above = 1;

None of the above = 0.

**The score of asbestos knowledge = sum of questions 1, 2, 3, 4, 5, 6, and 8.**
